# YOLOv8-ACU: improved YOLOv8-pose for facial acupoint detection

**DOI:** 10.3389/fnbot.2024.1355857

**Published:** 2024-02-01

**Authors:** Zijian Yuan, Pengwei Shao, Jinran Li, Yinuo Wang, Zixuan Zhu, Weijie Qiu, Buqun Chen, Yan Tang, Aiqing Han

**Affiliations:** ^1^College of Management, Beijing University of Chinese Medicine, Beijing, China; ^2^Beijing No.80 High School, International Department, Beijing, China; ^3^College of Acupuncture and Massage, Beijing University of Chinese Medicine, Beijing, China

**Keywords:** Chinese medicine acupoints, YOLOv8-pose, keypoint detection, ECA-net, slim-neck, GIoU

## Abstract

**Introduction:**

Acupoint localization is integral to Traditional Chinese Medicine (TCM) acupuncture diagnosis and treatment. Employing intelligent detection models for recognizing facial acupoints can substantially enhance localization accuracy.

**Methods:**

This study introduces an advancement in the YOLOv8-pose keypoint detection algorithm, tailored for facial acupoints, and named YOLOv8-ACU. This model enhances acupoint feature extraction by integrating ECA attention, replaces the original neck module with a lighter Slim-neck module, and improves the loss function for GIoU.

**Results:**

The YOLOv8-ACU model achieves impressive accuracy, with an mAP@0.5 of 97.5% and an mAP@0.5–0.95 of 76.9% on our self-constructed datasets. It also marks a reduction in model parameters by 0.44M, model size by 0.82 MB, and GFLOPs by 9.3%.

**Discussion:**

With its enhanced recognition accuracy and efficiency, along with good generalization ability, YOLOv8-ACU provides significant reference value for facial acupoint localization and detection. This is particularly beneficial for Chinese medicine practitioners engaged in facial acupoint research and intelligent detection.

## Introduction

Since the 1970s, WHO has been actively promoting acupuncture to the world, setting up acupuncture training institutions in many countries, supporting the creation of the World Federation of Acupuncture Societies (WFA), announcing many times the appropriate conditions for acupuncture treatment, encouraging global patients to choose acupuncture therapy, and striving to promote the internationalization and standardization of acupuncture (Lim, 2010). Acupuncture and massage are important parts of Chinese medicine, which is based on the theory of Chinese medicine's internal organs and meridians, with acupuncture and massage as the main treatment, and is an important means used to prevent and treat diseases and eliminate fatigue. By stimulating specific points on the human body, acupuncture and massage can regulate the body's qi and blood circulation and the balance of yin and yang, so as to achieve the purpose of treating diseases and relieving fatigue. Acupuncture and massage have been widely used in clinical practice and are gradually gaining attention and recognition worldwide. Acupoint localization serves as a pivotal component in the modalities of acupuncture and tuina therapy, where it's precision holds a direct correlation with the resultant therapeutic efficacy (Zheng, 2022). The imprecision in acupoint targeting may culminate in the ineffectuality of acupuncture interventions, potentially precipitating severe repercussions including, but not limited to, localized trauma, neural impairment, aneurysmal formations, ocular injuries, and incidences of needle fracture (Godson and Wardle, 2019). Pertaining to the human facial region, which is characterized by a plethora of acupoints each with distinct functionalities and in close proximity to one another, the task of exact acupoint identification is further complicated owing to the inter-individual anatomical variability, thereby presenting substantial challenges in accurate acupoint delineation (Lee et al., 2020). In clinical practice, the commonly used methods of taking acupoints can be divided into the method of anatomical marking on the body surface, the method of bone measurement and the method of body measurement (Zheng et al., 2005; Lin and Yi, 2019), all of which are artificial positioning of acupoints from the visual level, and the degree of accuracy is highly dependent on the professional skills and experience of physicians. Due to the complexity of human anatomy and individual differences, artificial positioning methods inevitably have certain subjectivity and errors, which affect the accuracy of acupoint positioning. Therefore, it is necessary to develop a new method to improve the objectivity, efficiency and accuracy of acupoint positioning to assist practitioners in prevention and treatment.

Artificial intelligence techniques may help to build intelligent, efficient and accurate models for point detection and localization. In recent years, AI techniques have been widely used in the fields of human behavior recognition, facial feature recognition and keypoint detection (Berretti et al., 2011; Maji et al., 2022; Pranavan et al., 2023; Zhang et al., 2023). However, as the available research deepens, the existing acupoint detection models have problems such as over-reliance on external devices such as infrared, insufficient mining of feature representations, and low accuracy and robustness of acupoint detection (Zhang et al., 2023). These issues seriously affect the generality of the models, and researchers are gradually recognizing the advantages of high-precision, lightweight detection models for better migration to mobile or embedded devices, and can achieve high-precision performance comparable to that of larger models.

Due to the problems of oversized models, low accuracy and insufficient robustness in existing studies, this study innovatively adopts the YOLOv8-ACU algorithm for facial acupoint recognition. Since acupoints do not have clear physical features or geometric shapes on the human surface, and locating facial acupoints by Chinese medicine practitioners is actually a process of locating facial regions or points, this study combines the facial acupoint detection task with face keypoint detection, and divides the task of facial acupoint detection into two aspects: identification of the type of acupoints and localization of the acupoints. Deep learning and computer vision techniques are used to recognize human facial features and acupoint location features, so as to achieve intelligent acupoint detection and assist doctors in acupoint treatment. The main contributions of this research are summarized as follows:

(1) The YOLOv8-pose model is applied to the self-constructed facial acupoints dataset and it has the advantages of high efficiency, speed, and accuracy compared with other models.
(2) The ECA channel attention mechanism is used to reduce the extraction of features outside the face, and is able to focus more on the extraction of facial acupoint features.
(3) Replacing the original neck module in YOLOv8-pose with Slim-neck can improve the recognition accuracy while lightening the weight.
(4) Replacing the loss function can more effectively improve the recognition effect and convergence speed of the model.

The algorithm used in this research has the advantages of high efficiency, multi-task processing, high accuracy, and robustness improvement, which better meets the needs of keypoint detection of facial acupoints. It possesses extremely important practical significance and clinical application potential. The specific implementation tools utilized in this algorithm are illustrated in Table 1.

**Table 1:** Implementing tools.

**Parameter**	**Type**
CPU	Intel(R) Core(TM) i9–11900H
Memory (GB)	16G
GPU	NVIDIA GeForce RTX 3060
Graphics Memory (GB)	6G
Training environment	CUDA 11.8 CUDNN 8.7
operating system	Windows 11 (64-bit)
development environment (computer)	Python3.10.11 Pytorch2.0.0

## Related works

In recent years, facial image processing, as a pivotal technology, has propelled technological advancements in domains such as facial recognition and image analysis. Deng et al. (2019) introduced a novel loss function, ArcFace, aimed at enhancing the capability of deep convolutional neural networks in feature learning. This innovation not only optimized the efficiency of feature extraction but also laid a foundational framework for the subsequent evolution of facial image processing technologies. Utilizing the efficient feature extraction methodology of ArcFace, Jin et al. (2022) applied deep learning models, originally developed for facial recognition tasks, to facial diagnostics. This cross-disciplinary application underscores the versatility and generalizability of deep learning models, offering new perspectives for medical image analysis and diagnostics. To further advance facial image processing, innovative techniques such as the generation of pseudo-depth information from traditional 2D RGB images using Generative Adversarial Networks (GANs) have been proposed (Jin et al., 2020). These advancements not only enhance the accuracy of facial recognition but also enable more complex facial recognition capabilities in resource-constrained settings. Concurrent with the rapid development of facial image processing, facial keypoint detection has emerged as a research focus, categorically divided into traditional facial keypoint detection methods and those based on deep learning. Among them, the traditional methods require manual design, feature extraction, and construction of subsequent classification or regression models, which can be further divided into parametric shape model-based methods and cascade shape regression-based methods. Among them, two representative algorithms based on parametric shape model approach are Active Shape Model (ASM) (Cootes et al., 1995), Active Appearance Models (AAM) (Cootes et al., 1998). ASM is a face keypoint detection algorithm proposed by Cootes et al. (1995), it is a point distribution model based algorithm, which firstly obtains the training set by manual calibration, and then obtains the shape model after training, and then abstracts the target object through the shape model, and then achieves the face shape matching through the keypoint matching in the testing stage. Cootes et al. (1998) further improved the ASM algorithm and obtained the AAM algorithm, which takes the texture features of the face region into account while adopting the shape constraints, and establishes the texture model while building the shape model, and combines the two models to obtain the active epistemic model. Dollár et al. (2010) put forward a classic work called the Cascaded Pose Regression (CPR), which is a method for matching the shape of the face. Cascaded Pose Regression model, which adopts a multi-stage cascade from coarse to fine learning idea to gradually improve the accuracy of keypoint locations through multiple iterations. This idea still influences many deep learning-based computer vision algorithms, such as Convolutional Pose Machines (CPM) (Wei et al., 2016) and Stacked Hourglass Network (Newell et al., 2016) in the field of human pose estimation, Cascaded R-CNN (Cai and Vasconcelos, 2018) in target detection and so on. Although the above traditional methods based on face keypoint detection can achieve better detection results to a certain extent, they inevitably have the limitations of complex data processing, poor anti-interference ability and low computational efficiency.

To overcome these problems, deep learning-based face keypoint detection methods have emerged in recent years and have substantially outperformed traditional methods in face keypoint detection tasks. Sun et al. (2013) first proposed and employed a deep convolutional neural network, DCNN (Deep convolutional network) (Ren et al., 2017), for face keypoint detection, proposing a three-stage coarse-to-fine cascade regression network, which achieved then-optimal results on several publicly available datasets. Currently, some scholars have applied deep learning-based keypoint detection techniques to face acupoint detection, and Zhang et al. (2023) constructed the face point dataset FAcupoint and proposed the FADbR facial point detection algorithm framework, which adequately extracts face features through the reconstruction task and reduces the model's dependence on labeled data. Berretti et al. (2011) conducted experiments on the BU-3DFE dataset to extract the local features of the face using the SIFT (Lowe, 2004) model for keypoint detection and used a multi-class SVM (Platt, 1998) for classification, and the experimental results showed that the method has a good performance in recognizing facial expressions. YOLO series algorithms, which use one-stage detection method, have many applications in keypoint detection by virtue of its simple network model and high accuracy rate. Maji et al. (2022) proposed the YOLO-pose algorithm for 2D multi-person pose keypoint estimation in images, achieving impressive results. On the MS COCO validation and test sets, the model achieved mAP@0.5 values of 90.2% and 90.3%, respectively. Pranavan et al. (2023) applied YOLOv7-pose to keypoint detection for assessing falls in elderly individuals, achieving an accuracy of 89.6% and precision of 91.2%, demonstrating good detection performance. However, the aforementioned models still have drawbacks such as slow speed, poor performance in detecting small faces, difficulties in training, and relatively low model accuracy. Therefore, this research proposes three improvements to the original YOLOv8-pose model, aiming to both reduce model size and enhance the accuracy of acupoint recognition.

ECA-net (Wang et al., 2020) is an improvement upon SE-net (Hu et al., 2018), introducing a non-dimensional reduction local cross-channel interaction strategy. This strategy effectively utilizes one-dimensional convolution and employs ResNets (He et al., 2016) and MobileNetV2 (Sandler et al., 2018) as backbones. The proposed ECA module has been extensively evaluated in image classification, object detection, and instance segmentation. Experimental results demonstrate that the module outperforms other modules in terms of performance while maintaining high efficiency. In this research, the ECA attention mechanism is employed to enhance the capture of acupoint features, with a focus on improving these features.

Slim-neck (Li et al., 2022) is used to balance model accuracy and speed. Originally applied to the SODA10M autonomous driving dataset, the Slim-neck detector shows significant improvements in speed and accuracy compared to the original detector. In this work, the Slim-neck module is substituted for the original neck module, enabling lightweight modeling while maintaining good detection performance.

GIoU (Rezatofighi et al., 2019) is an improvement upon IoU (Yu et al., 2016) and is utilized to optimize the non-overlapping regions. It has been verified that replacing IoU with GIoU significantly improves detection accuracy and is applicable to YOLO (Redmon et al., 2016) algorithms and the Faster R-CNN (Ren et al., 2015) series. In this research, GIoU is used as a loss function to enhance facial object detection capability and facial acupoint keypoint detection.

## Material and methods

This section firstly introduces the improvement process of YOLOv8-pose, and then gives a brief introduction to the YOLOv8 keypoint detection algorithm in subsection 3.1, explaining the role and structure of each module. In subsection 3.2, it focuses on the improvement strategy of this research and gives a detailed description of each improved module in subsections 3.2.1, 3.2.2, and 3.2.3.

**Figure 1:**
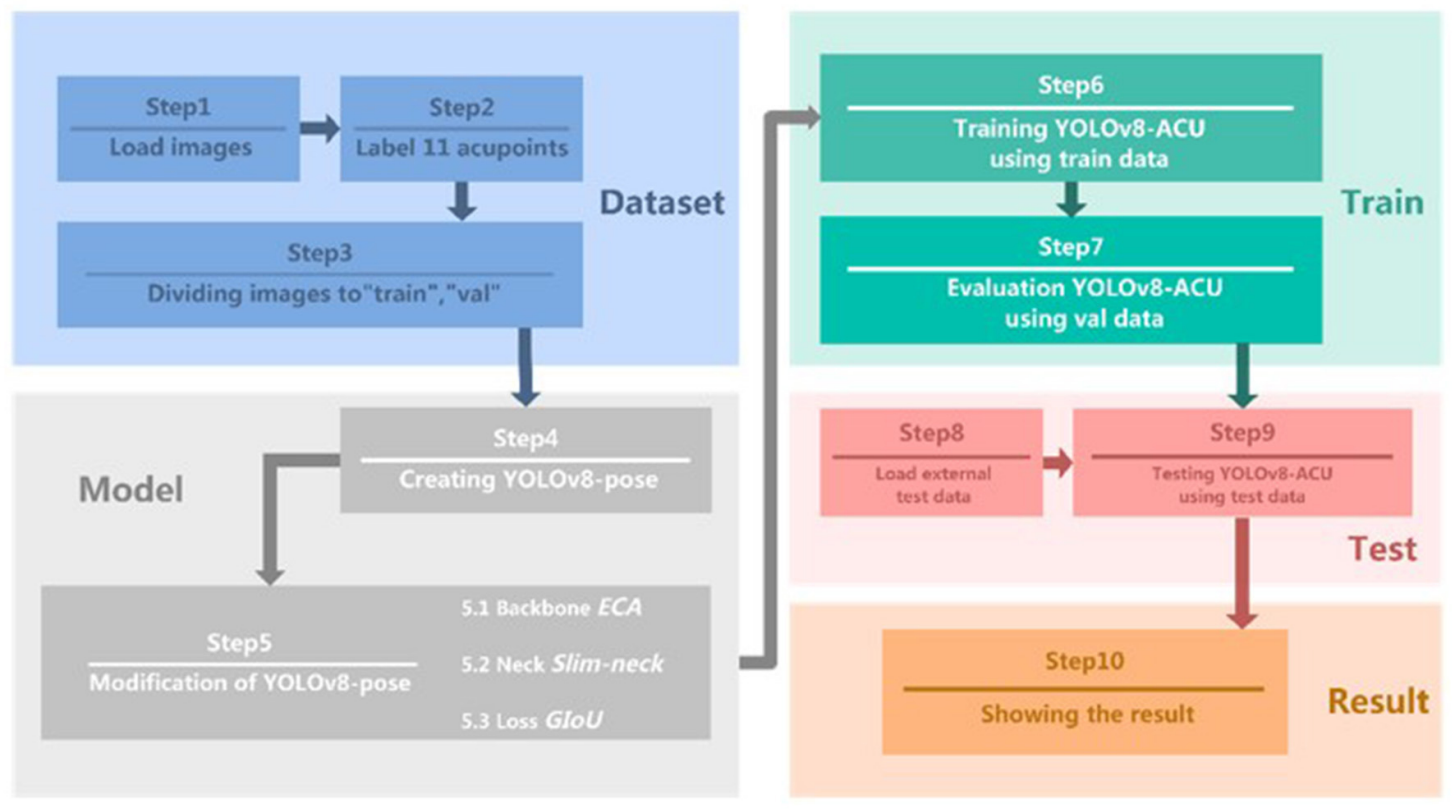
Experimental steps of keypoint detection model for acupoints.

The YOLOv8-ACU critical point detection model and the improved experimental flow proposed in this research are shown in Figure 1, including the following ten steps.

- Step 1: Select 608 front face photos from WIDER Face public dataset as the dataset for this experiment.
- Step 2: Use labelme software to manually label 11 types of acupoints.
- Step 3: Divide the dataset into training and validation sets.
- Step 4: Create an initial model of YOLOv8-pose for subsequent improvement of it.
- Step 5: YOLOv8-ACU model
  - Sub-step 5.1: Add ECA attention mechanism to the backbone part of the original model.
  - Sub-step 5.2: Replace the original model's neck module with a lighter weight Slim-neck.
  - Sub-step 5.3: Change the loss function to GIoU.
- Step 6: Fine-tune the original parameters in the model and train the data using YOLOv8-ACU.
- Step 7: Save the optimal model generated by the training process and evaluate the model performance on the validation set.
- Step 8: Collect additional facial image data as an external test set to complete the data labeling.
- Step 9: Apply external test set for model performance evaluation and the results are given in subsection 4.5.3.
- Step 10: Compare the evaluation results of the original model and the improved model detection, and output the model evaluation results.

### YOLOv8 keypoint detection algorithm

YOLOv8 (Jocher et al., 2020) is the latest detection algorithm introduced in the current YOLO series of algorithms, which is suitable for tasks such as target detection, image classification and instance segmentation. In this research, we focus on keypoint detection, and choose the smaller but more accurate YOLOv8n as the base model. The detection network of YOLOv8n is divided into four parts: Input, Backbone, Neck, and Head.

*Input:* The part is responsible for scaling the input image to the size needed for training, and carrying out data preprocessing and enhancement operations. Preprocessing includes normalization and scaling of images, ensuring consistency in the input size and pixel value range. For data enhancement, techniques such as Scaling, Tone Adjustment, Mosaic augmentation, and random transformations like cropping, rotating, and flipping are employed. The anchor-free mechanism is also adopted here to predict the center of the object directly, reducing the complexity and dependence on predefined anchor sizes and shapes.

*Backbone:* The backbone is used for feature extraction and contains Conv, C2f and SPPF modules. The new C2f structure applies residual features for learning, which enriches the gradient flow information; SPPF is called Spatial Pyramid Pooling, which converts an arbitrarily sized feature map into a fixed-size feature vector.

*Neck:* The structure of the neck follows the Feature Pyramid Network (FPN) (Lin et al., 2017) and the Path Aggregation Network (PAN) (Liu et al., 2018) effectively integrating the top-down and bottom-up information flow in the network and enhancing the detection performance.

*Head:* The head section utilizes different-sized feature maps to obtain category and position information for objects of varying sizes. It applies the concept of Distributional Focal Loss (DFL) (Qian et al., 2020), reducing the parameter size and computational complexity. The YOLOv8 series of models perform well in terms of detection accuracy and speed, and in this research, YOLOv8n is used as the basis for improvement to further enhance the detection performance.

### YOLOv8-ACU facial acupoint detection algorithm

**Figure 2:**
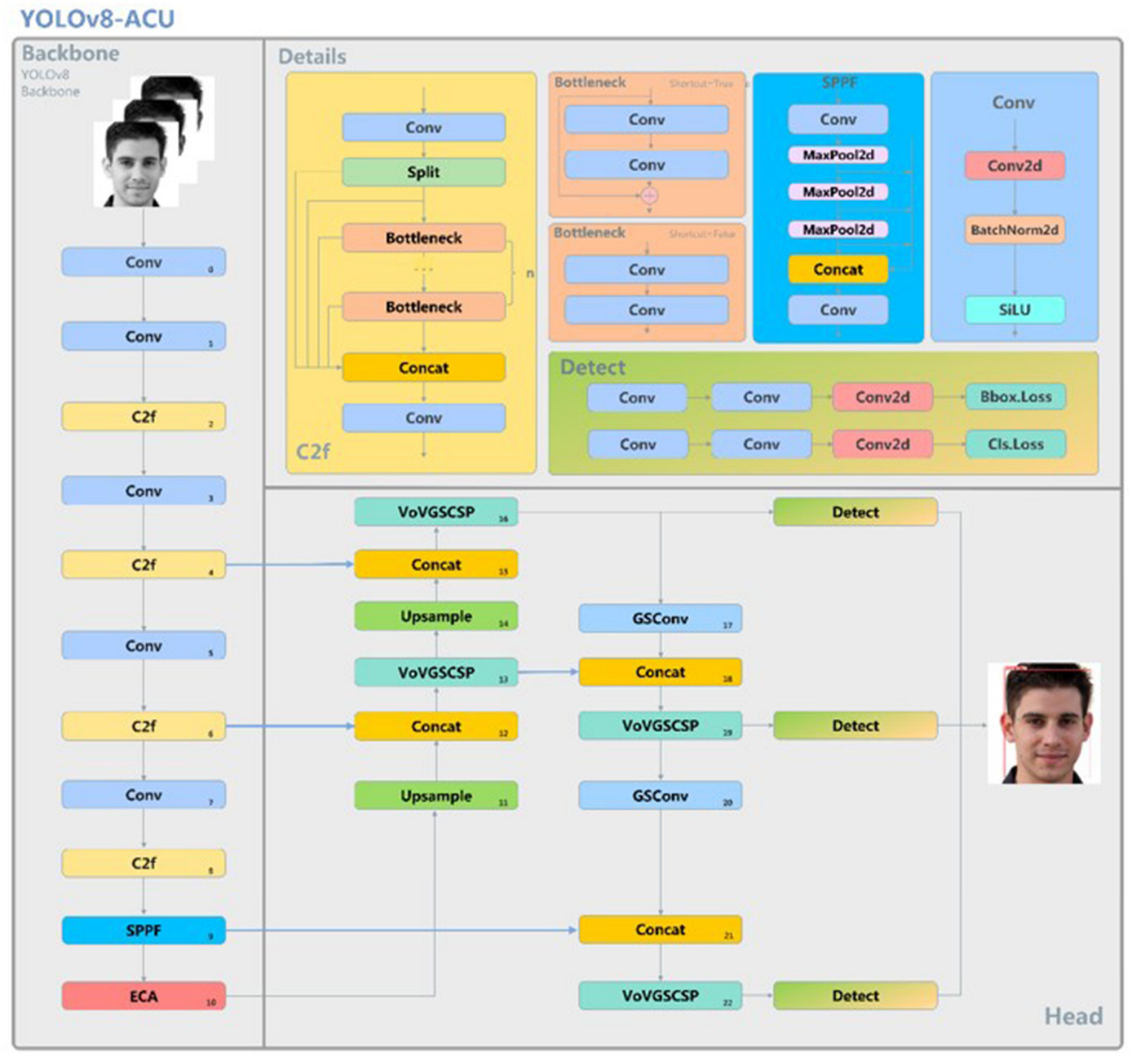
YOLOv8-ACU model structure.

The initial model of YOLOv8-pose is a keypoint detection model for human posture recognition. Since there are some differences in features between facial acupoint keypoint detection and human posture keypoint detection, and the detection of facial acupoints requires higher accuracy, this research improves the original YOLOv8-pose model, aiming to make it have the potential for clinical applications in the future. Because the distribution of facial acupoints is relatively dense, an effective ECA attention mechanism needs to be introduced into the feature extraction network of the backbone to enhance the feature representation capability and focus more attention on each acupoint, and the ECA attention mechanism is able to reduce the redundant computation while fully considering the global information, thus improving the performance and robustness of the model. Adopting Slim-neck instead of the original neck structure, by fusing the features of different layers, the network's ability to detect objects of different scales can be effectively improved, so as to reduce the detection error of the acupoints due to the change of the object's scale, and the Slim-neck can reduce the computational complexity of the model while improving the model's accuracy. Meanwhile, during the experiment, the loss function is further modified to use GIoU as the loss function. Its network structure is shown in Figure 2.

#### ECA attention mechanism

The main idea behind attention mechanisms is “dynamic weighting,” which assigns higher weights to important information and lower weights to relatively irrelevant information (Deng et al., 2023). As a new and efficient channel attention mechanism, the ECA attention mechanism mainly improves the SE attention mechanism, and achieves performance improvement through the local cross-channel interaction strategy without dimensionality reduction and adaptive selection of one-dimensional convolutional kernel size. Its specific steps are as follows:

- Step 1: Perform global average pooling operation on the input feature map
- Step 2: Conduct a 1D convolution operation with a kernel size of k, followed by a Sigmoid (Elfwing et al., 2018) activation function to obtain weights w for each channel.
- Step 3: The weights are multiplied with the corresponding elements of the original input feature map to obtain the final output features.

**Figure 3:**
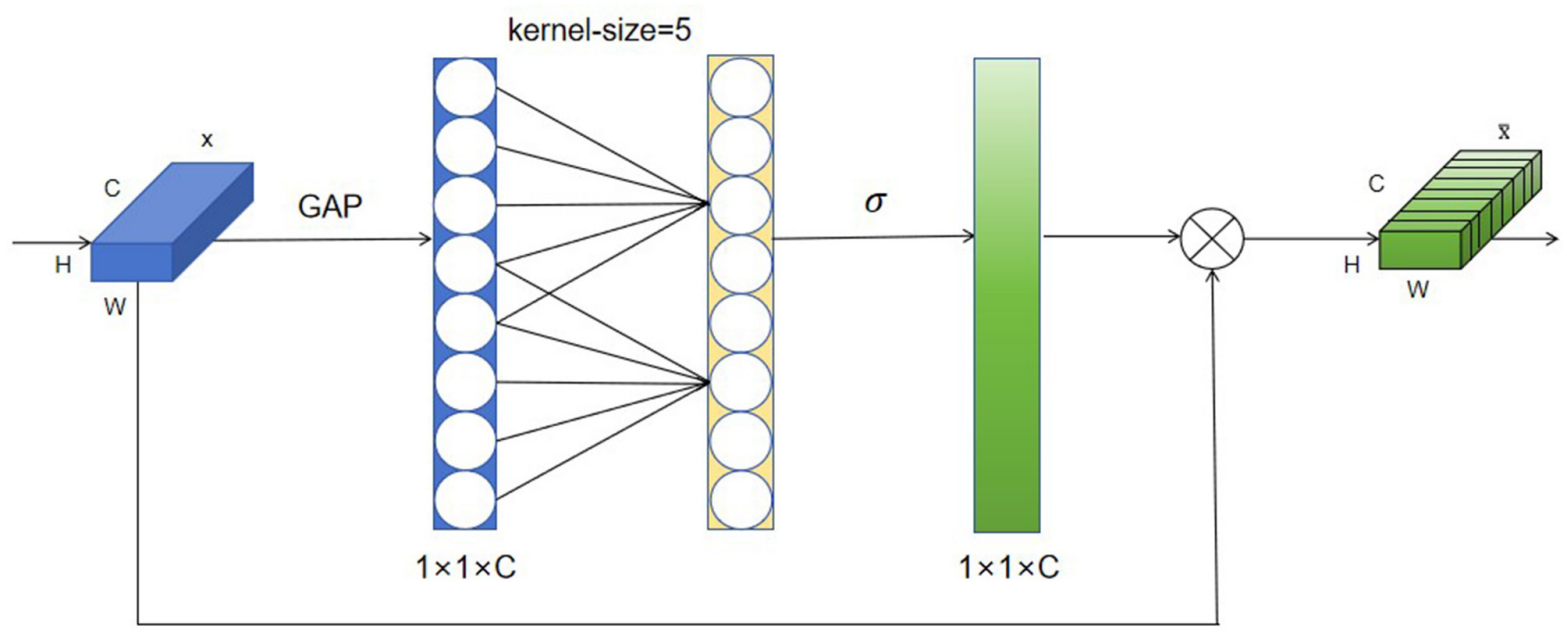
Flowchart of the ECA attention mechanism.

Incorporating the ECA attention mechanism into the backbone structure of YOLOv8-pose enables the neural network to pay more attention to the feature acquisition of acupoint locations and reduce the acquisition of other information beyond the face. Its flowchart is shown in Figure 3.

#### Slim-neck module

In order to accelerate the computation of the prediction, the input images in the convolutional neural network almost always need to undergo the following transformation process: the spatial information is gradually transferred to the channels, and each time the spatial (width and height) compression of the feature maps and the expansion of the channels lead to a partial loss of the semantic information, which makes the feature extraction for the acupuncture point recognition incomplete. Therefore, in this research, Slim-neck module is introduced to replace the original neck module in YOLOv8-pose in order to preserve as many hidden connections of each channel as possible.

**Figure 4:**
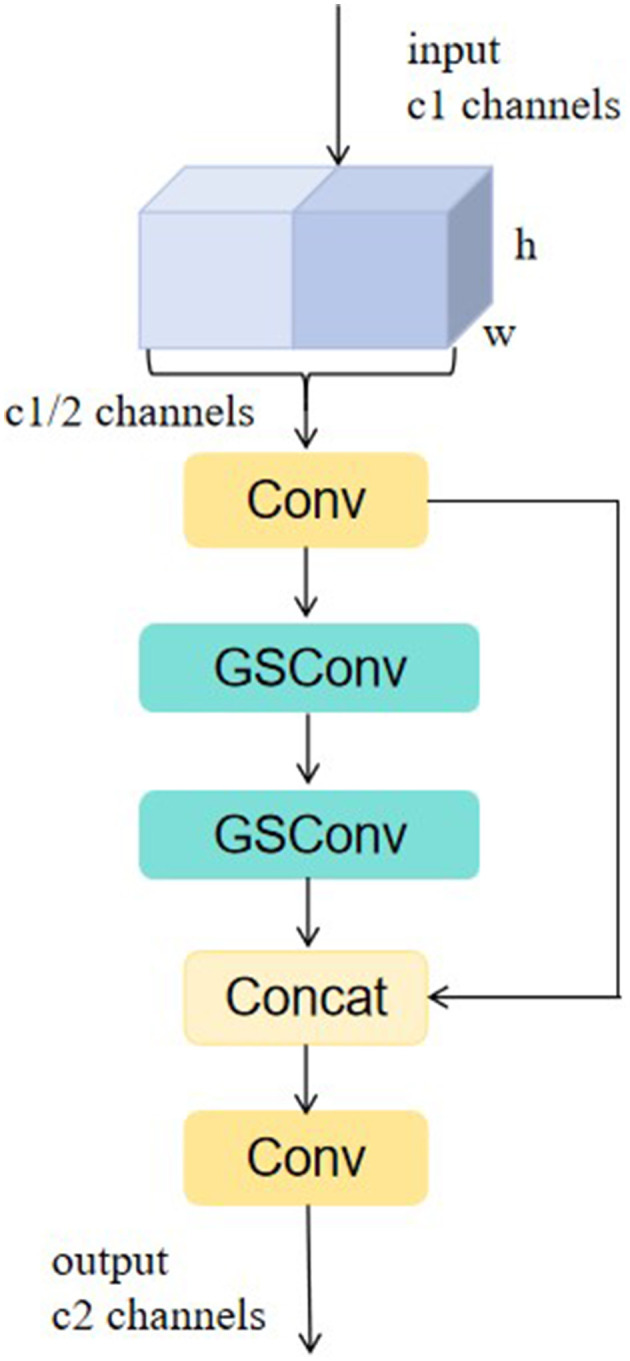
Structure of VoV-GSCSP.

Firstly, the traditional convolutional Conv is replaced in Slim-neck with the lightweight GSConv, a convolutional operation based on global pooling and group sparse concatenation, which improves the expressive power of the model while reducing the amount of computation by dividing the input channels into groups and performing independent convolutional operations on each group. The GSbottleneck module and VoV-GSCSP module are introduced on the basis of GSConv. The structure of VoV-GSCSP is shown in Figure 4. Among them, the VoV-GSCSP module uses the one-time aggregation method to design the cross-level partial network, and replaces the CSP module in the original neck with it, which reduces the amount of model computation and reduces the complexity of the network structure while maintaining sufficient accuracy. Slim-neck realizes the reconstruction of the neck module of YOLOv8-pose by combining GSConv and VoV-GSCSP, which is more conducive to the extraction and categorization of acupoint features, and also improves the accuracy of acupoint recognition while reducing the amount of model computation.

#### Improvement of the loss function

YOLOv8-pose fuses the target detection task and keypoint detection task together, so the accuracy of keypoint detection also depends on the accuracy of target detection to some extent, this research uses the GIoU loss function to replace the CIoU loss function on the target detection task (Zheng et al., 2020). The GIoU loss function, as an improved form of IoU, focuses not only on the overlapping region of the detection frame, but also on other non-overlapping regions, which can better reflect the overlap between the two. The formula for GIoU calculation is as follows:


(1) $$\begin{array}{l} GIoU=IoU-\frac{C-\left(B\cup B^{gt}\right)}{C} \end{array}$$

where C is the minimum circumscribed matrix area, B is the target prediction frame; *B*^*gt*^ is the ground-truth frame,according to Equation (1).

## Results

###  Facial acupuncture point detection dataset

In the research, experiments are conducted using Class I dataset and Class II dataset, where Class I dataset is used for training and validation of the model, while Class II dataset is used for testing of the model to evaluate and test the generalization ability of the model.

*Class I dataset:* The Class I dataset is constructed based on the publicly available WIDER Face dataset (Yang et al., 2016), selecting 608 images with frontal faces and limited occlusions for labeling 11 types of facial acupoints. To ensure the accuracy of annotation, three acupuncturists, each with over 20 years of extensive clinical experience and who have undergone regular training and updates, were invited to locate acupoints based on the International Standards of Acupuncture Points published by the WHO (World Health Organization Regional Office for the Western Pacific, 2008). In cases of disagreement, the principle of the minority conforming to the majority was adopted. If opinions were completely divergent, a consensus was reached through case study analysis and literature review discussions before proceeding with the localization. This process ultimately led to the formation of a Class I self-constructed dataset, Acupoint-I. During model training, the Acupoint-I dataset was divided into training and validation sets using an 8:2 ratio.

*Class II dataset:* The Class II dataset is an external dataset utilized by our team specifically for model testing. It consists of 236 frontal face photographs of diverse individuals, sourced both from the internet and the SCUT-FBP5500 public dataset. This dataset encompasses a balanced representation of adult males and females from both Asian and Caucasian ethnicities, with a deliberate 1:1 gender ratio maintained in the selection process. These images were annotated with acupoints by the three acupuncturists mentioned earlier, using the same methodology as that for the Class I dataset, leading to the formation of the self-constructed Class II dataset, Acupoint-II. Distinct from the model's training phase, the Class II dataset is exclusively deployed during the model evaluation stage. Its primary role is to accurately assess the model's generalization capabilities over unseen data and is not involved in the training process, ensuring no potential influence on the model's performance.

**Figure 5:**
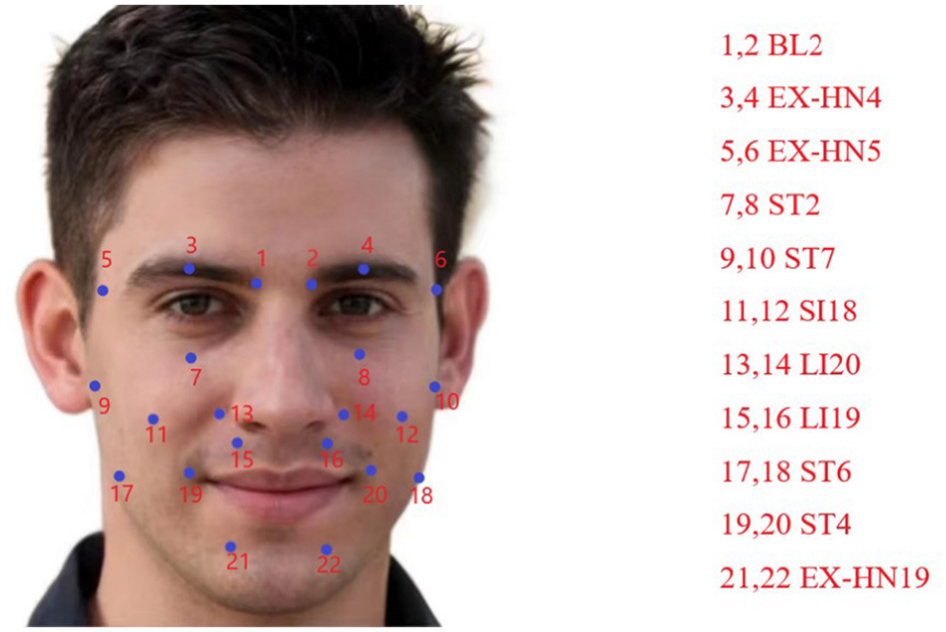
Introduction to acupuncture points.

*Introduction to acupuncture points:* In the world, acupuncture has become an indispensable part of daily medical practice and is now a widely used therapeutic modality in medicine (Chon and Lee, 2013). Following the guidance of the principle of syndrome differentiation and treatment, the selection of appropriate acupoints and their compatibility is a prerequisite for acupuncture treatment and a strong guarantee for improving clinical efficacy (Tian, 2010). Based on the experiences of Yu et al. (2021) and Zheng et al. (2009) in selecting acupoints for the treatment of peripheral facial paralysis, we finally selected Zanzhu (ST2), Sibai (ST7), Jiache (EX-HN19), Dicang (ST4), Taiyang (EX-HN4), and Quanliu (SI18) as main acupoints and Yuyao (EX-HN5), Yingxiang (LI20), Kouheliao (LI19), Jiachengjiang (ST6), and Jiache (EX-HN19) as complementary acupoints. The medical positioning of these 11 acupoints is as follows (as shown in Figure 5), according to the International Organisation for Standardization (2021):

- *Xiaguan (BL2):* Located in the depression between the central part of the lower edge of the zygomatic arch and the infraorbital margin.
- *Taiyang (EX-HN4):* Located at the depression approximately one horizontal finger's width (middle finger) posterior to the midpoint between the lateral end of the eyebrow and the outer canthus.
- *Yuyao (EX-HN5):* Located at the center of the eyebrow, directly above the pupil, with a tender sensation upon pressure.
- *Zanzhu (ST2):* Located in the depression at the medial end of the eyebrow, along the brow margin.
- *Sibai (ST7):* Located at the lower border of the zygomatic arch.
- *Quanliu (SI18):* Located in the depression below the zygomatic bone and directly below the lateral canthus.
- *Yingxiang (LI20):* Located at the side of the face, beside the midpoint of the nasolabial groove.
- *Kouheliao (LI19):* Located at the outer edge of the nostril, one-third of the distance from the upper to the lower part of the philtrum groove.
- *Jiachengjiang (ST6):* Located at a point one inch away on each side of the midpoint of the mental groove, parallel to Jiacheng (ST6).
- *Dicang (ST4):* Located at the side of the mouth, 0.4 cun (1 cun ≈ the width of the middle joint of the thumb) away from the corner of the mouth.
- *Jiache (EX-HN19):* Located approximately one horizontal finger's width (middle finger) above the anterior angle of the mandible.

### Comparison with previous works

*Comparison with YOLO-pose:* YOLO-pose is improved based on YOLOv5 model and its basic structure is similar to YOLOv5. In the backbone of YOLOv8-ACU model, the CSP idea is still adopted. In order to achieve further lightweighting of the model, the C2f module is introduced to replace the C3 module in YOLOv5. In addition despite some architectural optimization and improvements in YOLOv8, it still retains the SPPF module in the YOLOv5 architecture, which helps to enhance the feature extraction capability of the model.

**Figure 6:**
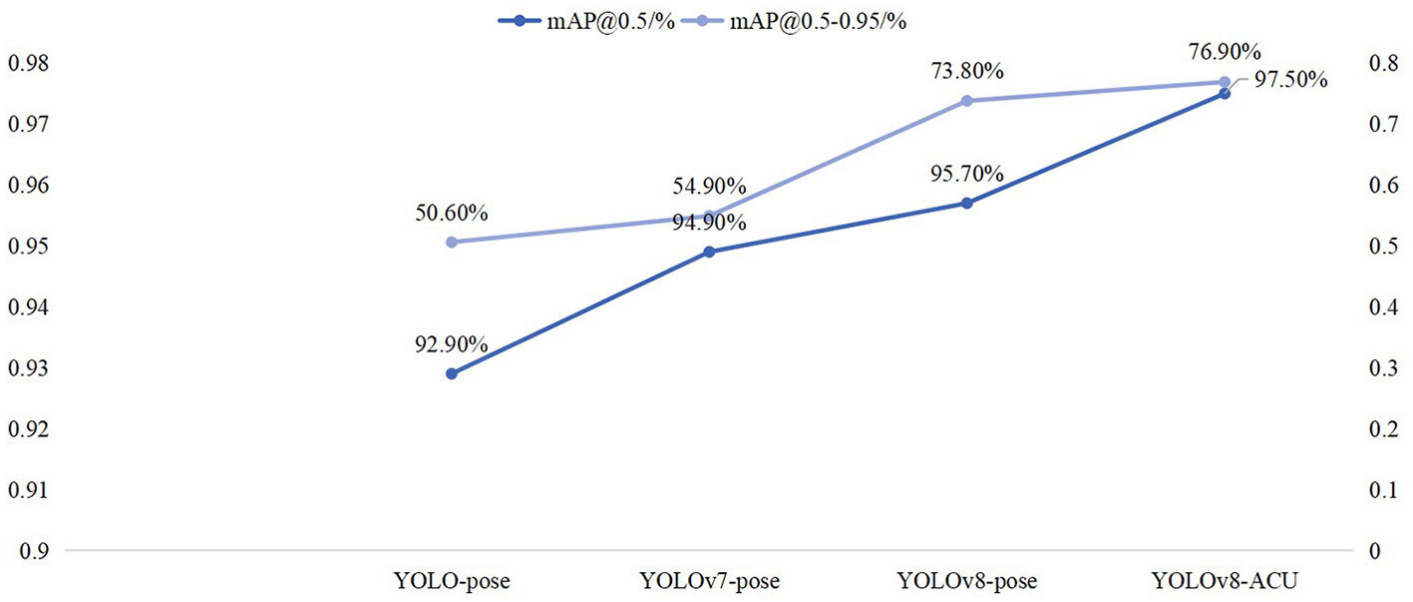
Comparison of mAP@0.5 and mAP@0.5–0.95 results for four models.

YOLOv5 adopts the Coupled head + Anchor-based approach for the head part, while YOLOv8 utilizes the Decoupled head + Anchor-free approach. As a result, YOLOv8's decoupling of the detection head from the anchor boxes enhances the adaptability to objects of different scales, leading to improved detection performance (as shown in Figure 6).

Experimental results have shown that, with a batch size of 4, the YOLO-pose model achieves a default configuration and initial parameters that results in mAP@0.5 value of 92.90% for acupoint keypoint detection task. Under the same settings, YOLOv8-pose, with the application of AdamW, achieves an mAP@0.5 value of 95.70%. Furthermore, the parameter count of YOLOv8-ACU reduces from 7.2M in YOLO-pose to 2.96M, while the computational load decreases from 16.8 GFLOPs in YOLO-pose to 8.8 GFLOPs in YOLOv8-ACU. This indicates that YOLOv8-ACU not only improves the detection performance, but also makes the model more lightweight and efficient.

*Comparison with YOLOv7-pose:* The C2F module in YOLOv8 is inspired by the E-ELAN module in YOLOv7, which enhances the gradient flow in the model by fusing different feature maps from multiple branch cross-layer networks. YOLOv8 achieves lightweight modeling by utilizing depth-wise separable convolution layers to reduce the parameter size and computational load.

In our experimental setup, when the batch size is set to 4, applying YOLOv7-pose for acupoint keypoint detection yields an mAP@0.5 of 94.9%. However, under the same settings, YOLOv8-pose achieves an mAP@0.5 of 97.5%.

Alongside the improvement in accuracy, YOLOv8-ACU significantly reduces the parameter count from 80.3M in YOLOv7 to 2.96M, a reduction of nearly 26 times, while the computational load decreases from 101.8GFLOPs to 8.8GFLOPs, a decrease of 91%. Considering the overall detection performance and model complexity, YOLOv8-ACU maintains a high mAP value while experiencing a significant reduction in parameters and computational load, making it more suitable for real-world deployment. Therefore, compared to YOLOv7-pose, YOLOv8-ACU achieves both accuracy and efficiency improvements in acupoint keypoint detection tasks, making it more applicable in practical scenarios (as shown in Figure 6).

### Implementing tools and model parameters

The training iteration period is set to 75, the batch size is set to 4, the optimiser is chosen to be AdamW, the initial learning rate is set to 0.01, and the momentum factor to 0.937.

### Evaluation metrics

In the research, P (Precision) and R (Recall) are used as evaluation indexes for target detection. The calculation method of the indexes is as follows.

Precision is the proportion of positive class targets detected by the model that are truly positive class targets. In target detection, precision represents the ratio of targets correctly detected by the model to all bounding boxes detected as targets by the model. The calculation formula is Equation (2):


(2) $$\begin{array}{l} P=\frac{TP}{TP+FP} \end{array}$$

Recall is the ratio of positive class targets detected by the model to the actual positive class targets, according to Equation (3). In target detection, the recall rate represents the ratio of targets correctly detected by the model to the bounding boxes of all true positive class targets.


(3) $$\begin{array}{l} R=\frac{TP}{TP+FN} \end{array}$$

In keypoint detection, this study uses the official MS COCO given based on the object keypoint similarity *L*_*oks*_ (object keypoint similarity) (Maji et al., 2022) The average accuracy mean of the validation criteria is used as the evaluation metric. Where *L*_*oks*_ is denoted as Equation (4):


(4) $$L_{oks}=\frac{\sum_{i}\left[\exp^{\left(-\frac{d_{i}^{2}}{2s^{2}k_{i}^{2}}\right)}\delta \left(v_{i}>0\right)\right]}{\sum_{i}\delta \left(v_{i}>0\right)}$$

where *i* is the labeled keypoint number;$d_{i}^{2}$ is the square of the Euclidean distance between the detected keypoint location and the true keypoint location;*s*^2^ is the area occupied by the detected body in the image;*k*_*i*_ is the decay constant used to control the keypoint category*i* of the attenuation constant;δ is the impulse function, indicating that it computes the value only for the visible keypoints *L*_*oks*_ in the true annotations;*v*_*i*_ is the value of the visibility of the *i*-th keypoint (*v*_*i*_>0 indicates that the keypoint is visible).


(5) $$\begin{array}{l} Precision_{kpt}=\;\frac{TP_{kpt}}{TP_{kpt}+FP_{kpt}} \end{array}$$

(6) $$\begin{array}{l} Recall_{kpt}=\;\frac{TP_{kpt}}{TP_{kpt}+FN_{kpt}} \end{array}$$

(7) $$AP=\;\int_{0}^{1}Precision_{kpt}dRecall_{kpt}$$

(8) $$mAP=\;\frac{\sum_{i=1}^{N}AP_{i}}{N}$$

*TP*_*kpt*_(True Positives) occurs when acupoints are correctly identified, with their predicted keypoints showing an *L*_*oks*_ above the threshold in comparison to actual acupoint keypoints. *FP*_*kpt*_(False Positives) arises when non-acupoint areas are incorrectly identified as acupoints, indicated by predicted keypoints exceeding the *L*_*oks*_ threshold. *FN*_*kpt*_(False Negatives) represents situations where actual acupoint keypoints are missed because the corresponding predicted keypoints do not meet the required *L*_*oks*_ threshold. The AP value is the area of the P-R curve according to Equation (5)-(7). mAP@0.5 is the average of the AP values for all categories at a threshold of 0.5. mAP@0.5–0.95 considers a range of different *L*_*oks*_ thresholds, from 0.5 to 0.95, at intervals of 0.05 (for example, 0.5, 0.55, 0.6, …, 0.95), and calculates the average of the AP values at these varying thresholds according to Equation (8).

Additionally, this study selects the GFLOPs and the number of Parameters as evaluation criteria to measure the size of the model.

### Experimental results and analysis

#### Comparative experiments on keypoint detection models

To assess the comparison between YOLOv8-pose and other keypoint detection models, this study selected four different models for comparative experiments: YOLO-pose, YOLOv7-pose, YOLOv8-pose, and YOLOv8-ACU. The experimental results for each model are shown in the table above. From the experimental results, it was found that when using pretrained models, YOLO-pose achieved 92.90% in mAP@0.5 and 50.60% in mAP@0.5–0.95, while YOLOv7-pose achieved 94.90% in mAP@0.5 and 54.90% in mAP@0.5–0.95. It can be observed that both YOLO-pose and YOLOv7-pose perform exceptionally well in terms of accuracy, but their model parameters and computational complexity are quite high.

On the other hand, YOLOv8-ACU outperforms other models in various aspects. Even when YOLO-pose and YOLOv7-pose are equipped with pretrained weights, they still cannot match the keypoint prediction accuracy of YOLOv8-ACU. Moreover, in clinical applications of facial acupoint detection, a higher recognition accuracy is typically required. Therefore, after incorporating Slim-neck, ECA attention mechanism, and more suitable loss functions, YOLOv8 achieved a 1.8% improvement in mAP@0.5 and a 3.1% improvement in mAP@0.5–0.95 compared to the original YOLOv8-pose. These improvements represent significant advancements in accuracy. Additionally, as shown in Table 2, YOLOv8-ACU has the smallest number of parameters and computational complexity among these four models, making its lightweight design more suitable for real-world clinical practice.

**Table 2:** Comparative experiments.

**Model**	**Pre-trained**	**mAP@0.5/%**	**mAP@0.5–0.95/%**	**Parameters**	**GFLOPs**
YOLO-pose	Yes	92.90%	50.60%	7231912	16.8
YOLOv7-pose	Yes	94.90%	54.90%	80293736	101.8
YOLOv8-pose	No	95.70%	73.80%	3403229	9.7
YOLOv8-ACU	No	97.50%	76.90%	2962560	8.8

#### Ablation experiment

**Figure 7:**
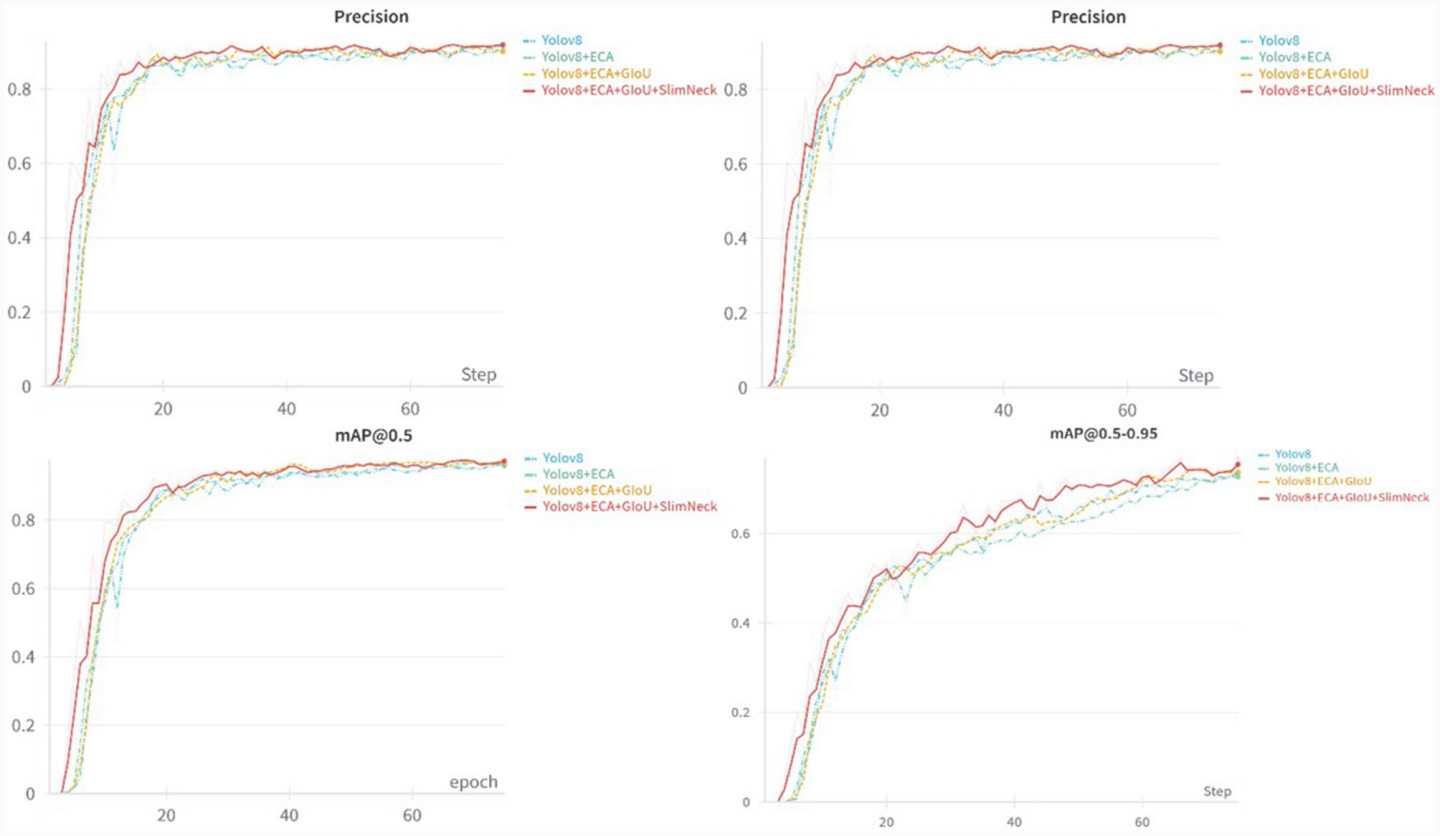
Comparison of results from precision, recall, mAP@0.5, and mAP@0.5–0.95.

**Figure 8:**
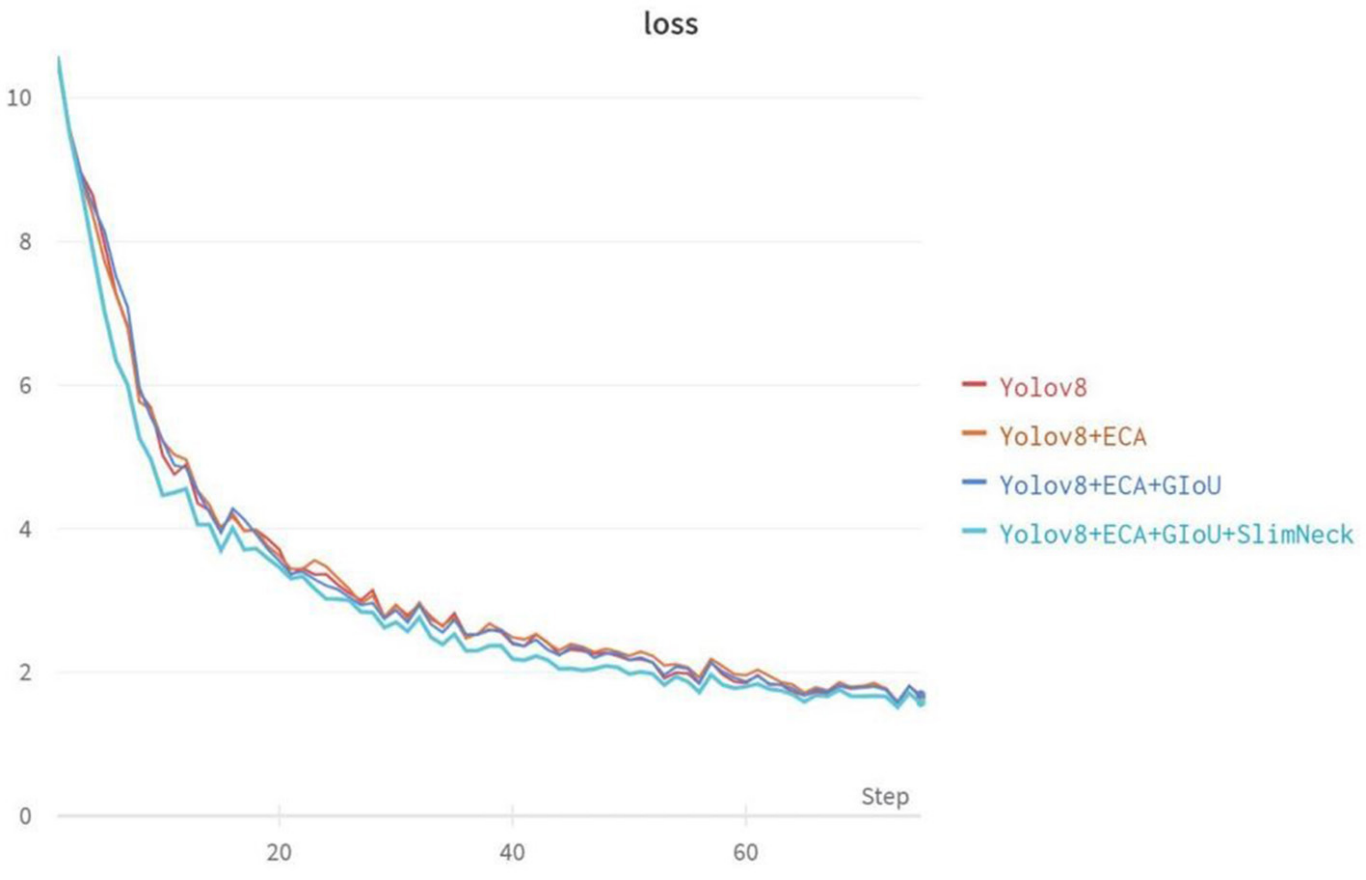
Variation curves of loss function for ablation experiments.

According to the ablation experiments in Figure 7, it can be observed that adding the ECA attention mechanism improved the Recall by 3.1% and mAP@0.5 by 0.9%. Adding the GIoU loss function increased the Recall by 0.2%, mAP@0.5 by 0.2%, and mAP@0.5–0.95 by 1.4%. Integrating the lightweight structure, Slim-neck, improved Precision by 2.2%, mAP@0.5 by 0.7%, and mAP@0.5–0.95 by 2.9%. Additionally, the model Parameters, Model Size, and GFLOPs decreased by 0.44M, 0.82MB, and 9.3%, respectively. From the loss function variation curve in Figure 8, it can be observed that changing the loss function to GIoU Loss resulted in faster model convergence and improved accuracy.

The experimental results in Table 3 demonstrate that compared to the original YOLOv8-pose network model, the proposed YOLOv8-ACU model achieved improvements in Precision (+0.2%), Recall (+2.0%), mAP@0.5 (+1.8%), and mAP@0.5–0.95 (+3.1%). Additionally, the Parameters, Model Size, and GFLOPs of the model decreased by 0.44M, 0.82MB, and 9.3%, respectively, validating the effectiveness of the proposed algorithm improvements.

**Table 3:** Ablation experiment.

**ECA**	**GIoU**	**SlimNeck**	**Precision /%**	**Recall /%**	**mAP@0.5 /%**	**mAP@0.5–0.95/%**	**Parameters /M**	**ModelSize /MB**	**GFLOPs**
X	X	X	92.0	95.0	95.7	73.8	3.40	6.87	9.7
✓	X	X	90.8	98.1	96.6	72.6	3.40	6.87	9.7
✓	✓	X	90.0	98.3	96.8	74.0	3.40	6.87	9.7
✓	✓	✓	92.2	97.0	97.5	76.9	2.96	6.05	8.8

#### Verification of external test sets

To test the improved model's generalization ability and effectiveness in clinical applications, in the research, we employed an external test set consisting of frontal face photos in approximate real-world scenarios for independent model validation. The model, as shown in Table 4, achieved Precision of 99.6%, Recall of 99.8%, mAP@0.5 of 99.5%, and mAP@0.5–0.95 of 80.7%, surpassing the performance on the Class I dataset. This demonstrates that the improved model exhibits strong generalization capabilities when handling real-world data, which is crucial for its application in actual clinical environments. In specific clinical scenarios where physicians are involved, the model must accurately identify frontal face photos to support acupuncture or massage therapies. Therefore, the improved model can effectively locate 11 facial acupoints and assist healthcare professionals in intelligent acupoint recognition during clinical practice.

**Table 4:** Test set validation results.

**Precision/%**	**Recall/%**	**mAP@0.5/%**	**mAP@0.5:0.95/%**
99.6	99.8	99.5	80.7

#### Acupoint detection results

**Figure 9:**
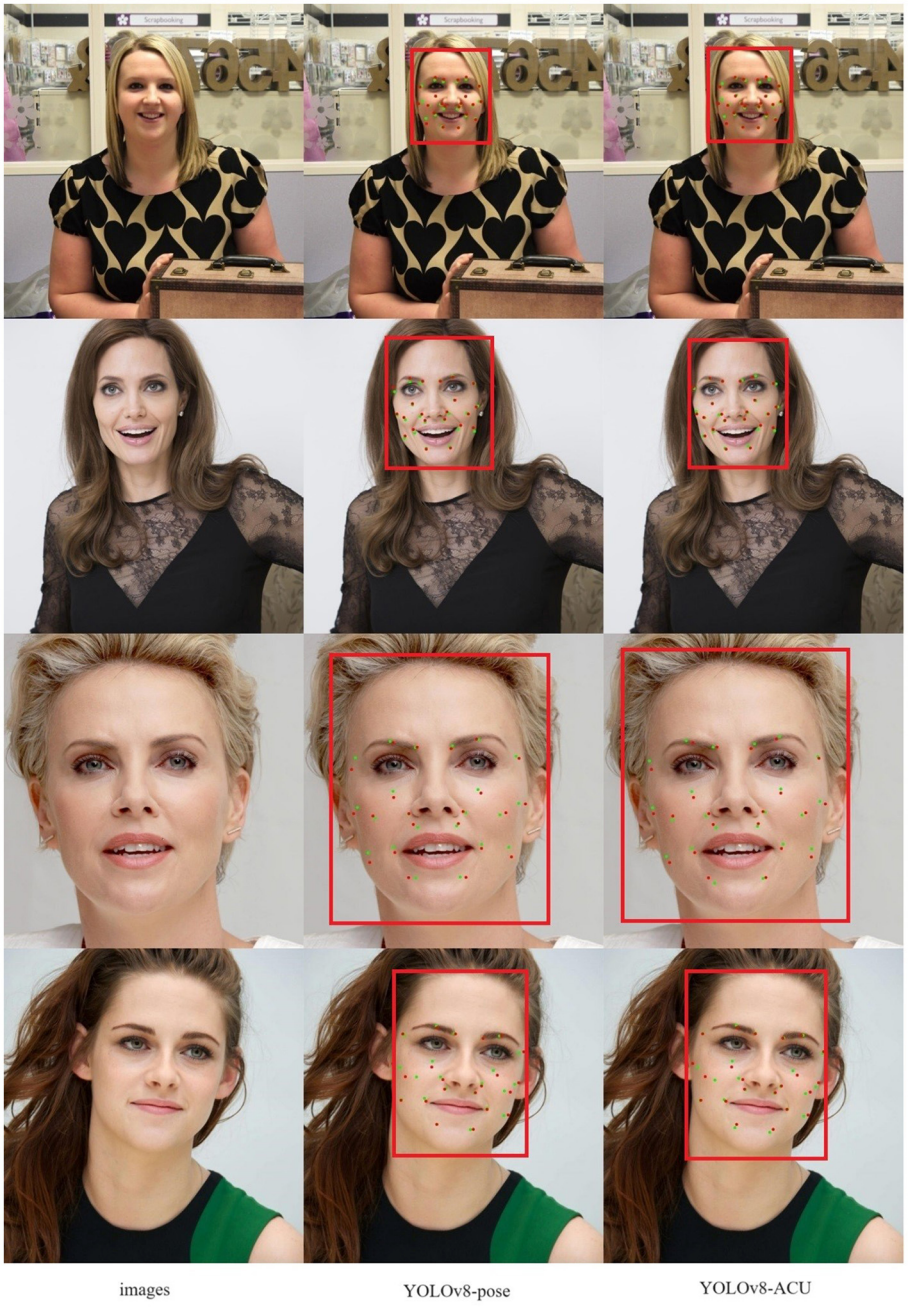
Acupoint-I acupoint prediction results.

**Figure 10:**
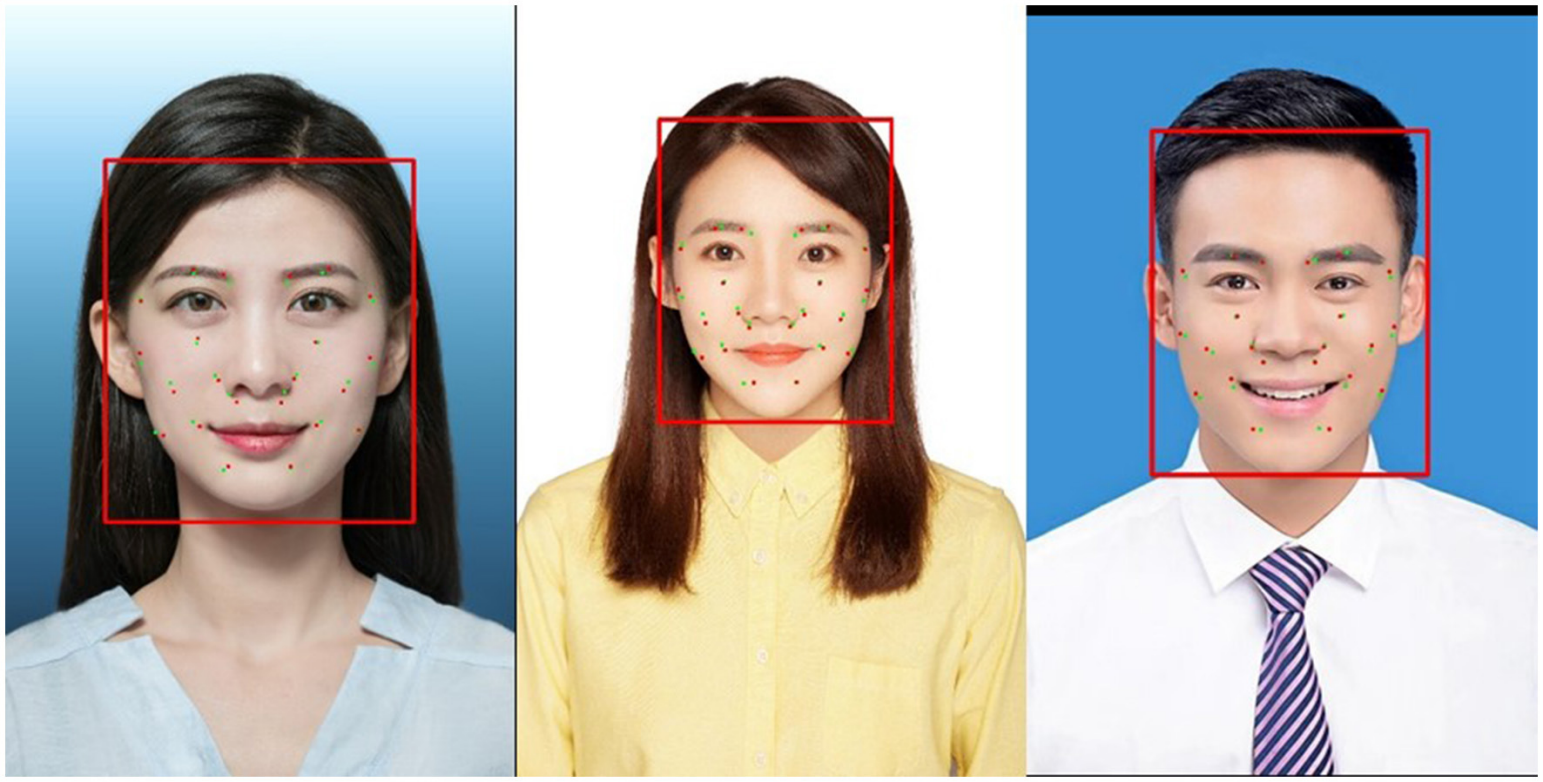
Acupoint-II acupoint prediction results.

As shown in Figure 9 (red dots indicate predicted acupoint locations and green dots indicate true acupoint locations; it is important to note that subtle differences in the sizes of the acupoint dots are due to the varying resolutions of the images), through the comparison of the detection effects of YOLOv8-pose and YOLOv8-ACU, it can be found that the detection effects of YOLOv8-ACU and YOLOv8-pose on acupoints both present a good level of recognition, and most of the detection effects of YOLOv8-ACU on acupoints are better than that of YOLOv8-pose, and the relative positions of the predicted acupoints and the actual ones are also closer. Positions are also closer to each other as shown in Figure 10. Figure 10 shows the results of the validation of YOLOv8-ACU on the images in the external dataset, since the images in the external test set are closer to the actual clinical environment, and the imaging of the human face is clearer and more obvious, the detection of this part of the images using YOLOv8-ACU can have an extremely strong recognition and detection ability, and the predicted acupoint positions are more closely matched to the actual acupoint positions. However, due to a variety of factors such as sample size and individual differences, the model still has some error and uncertainty, and there is still room for improvement in recognition accuracy and precision. In the future, the acupoint detection method will be further improved to enhance the accuracy and reliability and ensure the applicability in different environments and populations.

## Discussion

Acupuncture points are a key concept in Chinese medicine, different points have different effects, and the precise identification of points is also the most important part of the acupuncture therapy process (Li et al., 2015). There are fewer studies applying keypoint detection models to acupoint recognition, Zhang et al. (2023) used the FADbR model to detect the acupoints on the face, and most of the acupoints are detected well, but the detection effect is poor in the sparse position of the acupoints, and the model only has the ability of keypoint detection, while YOLOv8-ACU has the ability of both the target detection and the keypoint detection, which is able to have a better interactivity, and the applicable scenarios are more abundant. In comparison with YOLO-pose (Maji et al., 2022) and YOLOv7-pose (Pranavan et al., 2023) YOLOv8-ACU can also be found to have better accuracy and lighter model size.

By comparing YOLOv8-ACU, YOLOv8-pose, YOLOv7-pose, YOLO-pose, it can be found that YOLOv8-ACU not only shows better results in accuracy, but also is lighter in model size, and has a good generalization ability in the external test set. It shows that the recognition effect and performance of the model can be effectively improved by adding the ECA attention mechanism, using the Slim-neck module and changing to the GIoU loss function.

However, the model also has some defects. There are fewer categories of acupoints, which do not cover all the acupoints on the face, and there is no classification experiment for data under various light and occlusion conditions, which may lead to errors in the detection and localization of acupoints under low light, dark light or occlusion conditions. In the future, we will further expand the categories of facial acupoints and improve the performance of detecting acupoints under different occlusions and different light conditions, and deploy them in embedded devices for real clinical practice.

## Conclusions

To enhance the accuracy of facial acupoint recognition for better application in clinical practice, this study utilized the YOLOv8-pose model, which has been recently introduced with high detection and localization accuracy, as the base model. It was applied to the task of facial acupoint recognition. Building upon this base model, the study incorporated the ECA attention mechanism, introduced the Slim-neck module, and replaced its loss function. These enhancements further improved the precise recognition of acupoints while reducing the model's complexity and computational load. Compared to the base YOLOv8-pose model, YOLOv8-ACU demonstrated improvements in Precision (+0.2%), Recall (+2.0%), mAP@0.5 (+1.8%), and mAP@0.5–0.95 (+3.1%). Additionally, the Parameters, Model Size and GFLOPs decreased by 0.44M, 0.82MB, and 9.3% respectively. Comparing with other keypoint models, YOLOv8-ACU emerged as the most suitable model for acupoint recognition.

## Data availability statement

The raw data supporting the conclusions of this article will be made available by the authors, without undue reservation.

## Ethics statement

Written informed consent was obtained from the individual(s) for the publication of any potentially identifiable images or data included in this article.

## Author contributions

ZY: Conceptualization, Data curation, Formal analysis, Funding acquisition, Investigation, Methodology, Project administration, Resources, Software, Supervision, Validation, Visualization, Writing – original draft, Writing – review & editing. PS: Formal analysis, Funding acquisition, Methodology, Writing – original draft, Writing – review & editing, Project administration, Resources. JL: Conceptualization, Data curation, Formal analysis, Funding acquisition, Writing – original draft. YW: Investigation, Methodology, Project administration, Supervision, Writing – original draft, Writing – review & editing. ZZ: Data curation, Writing – original draft. WQ: Data curation, Writing – original draft. BC: Data curation, Writing – original draft. YT: Resources, Software, Supervision, Validation, Writing – original draft, Writing – review & editing. AH: Conceptualization, Software, Supervision, Validation, Visualization, Writing – original draft, Writing – review & editing.
